# Herausforderungen für die Lehrkräftefortbildung vor dem Hintergrund der digitalen Transformation – Perspektiven der Erwachsenen- und Weiterbildung

**DOI:** 10.1007/s40955-022-00212-y

**Published:** 2022-06-14

**Authors:** Caroline Bonnes, Johannes Wahl, Andreas Lachner

**Affiliations:** 1 Universität Konstanz, Konstanz, Deutschland; 2 Eberhard Karls Universität Tübingen, Tübingen, Deutschland

**Keywords:** Lehrkräftebildung, Lehrkräftefortbildung, Digitale Transformation, Medienpädagogische Kompetenz, Fortbildung, Professionalisierung, Teacher training, Continuing teacher training, Digital transformation, Media-pedagogical competence, Further education, Professionalization

## Abstract

Angesichts der digitalen Transformation schulischer Kontexte ergeben sich neue Anforderungen an die berufliche Handlungskompetenz von Lehrkräften. Dies führt zu einem erhöhten Handlungsbedarf im Bereich der Lehrkräftefortbildung. Diese befindet sich als eine spezifische Form beruflicher Fortbildung an der Schnittstelle zwischen Lehrkräftebildung und Erwachsenen- und Weiterbildung, so dass eine Betrachtung dieser Lernkontexte aus dieser Perspektive subdisziplinübergreifende Impulse für die Umsetzung und Gestaltung von Lehrkräftefortbildung geben kann. Im Beitrag werden daher aktuelle Herausforderungen der Lehrkräftefortbildung vor dem Hintergrund der digitalen Transformation anhand des Mehrebenensystems der Erwachsenen- und Weiterbildung aufgezeigt und mit Blick auf die Erkenntnisse und Konzepte der Erwachsenen- und Weiterbildung diskutiert.

## Abstract (en)

In the face of the ongoing digital transformation within the school context, new challenges arise for the professional competence of teachers. This leads to an increased need for action within continuing teacher training which is closely connected to the discipline of adult and further education. Examining the context of further teacher training through this disciplinary lense allows for cross-field impulses for its implementation and design. In this article we depict present challenges for continuing teacher education on the basis of the multi-level governance of adult and further education. These challenges will be discussed in view of findings and concepts of adult and further education.

## Einleitung

Die digitale Transformation (Schrape 2021) und die damit verbundene zunehmende Präsenz von digitalen Medien und ihren (Infra‑)Strukturen führt in allen Bereichen des Bildungssystems und damit auch in schulischen Kontexten zu vielfältigen Veränderungen. Im Zuge dieses Prozesses der sukzessiven „Verfestigung neuartiger soziotechnischer Prozesszusammenhänge durch die soziale Aneignung digitaltechnischer (Infra‑)Strukturen und die damit verknüpfte Rekonfiguration gesellschaftlicher Ordnungsmuster“ (Schrape 2021, S. 87) lassen sich auch Auswirkungen auf die Anforderungen an die Beschäftigten diagnostizieren (Eickelmann et al. 2019b). Spätestens durch die Strategie der Kultusministerkonferenz „Bildung in der digitalen Welt“ (KMK 2016) wurde das Thema auch bildungspolitisch stärker in den Vordergrund gerückt. Schülerinnen und Schüler sollen auf ein Leben in einer digitalisierten Welt vorbereitet werden. Dies setzt voraus, dass Lehrkräfte über die entsprechenden Kompetenzen verfügen. Sie müssen digitale Medien didaktisch sinnvoll verwenden, digitalisierungsbezogene Fachinhalte in ihren Unterricht integrieren und auf der Ebene der Medienerziehung und -bildung die Kompetenzen der Schülerinnen und Schüler im reflektierten Umgang mit digitalen Medien in einer sich wandelnden Gesellschaft fördern können.

Die digitale Transformation mit ihrer spezifischen sektoralen Eingriffstiefe in die Institution Schule und die Lebenswelt von Schülerinnen und Schülern, Erziehungsberechtigten und Lehrkräften bildet einen wesentlichen Anlass für Professionalisierungsprozesse von Lehrkräften in allen Stadien ihrer Berufsbiografie. Im Kontrast zu anderen gesellschaftlichen Herausforderungen bspw. im Bereich der Inklusion oder Nachhaltigkeit zeigt sich ihr transformativer Charakter in dem Umstand, dass die entsprechenden Veränderungen ausgehend von deutlichen technischen Innovationen auch ohne prospektive gesellschaftliche Diskussion zu Veränderungen gesellschaftlicher Interaktionsmodi führen. Die angesichts dieser Konstellation notwendigen Lernprozesse aus den drei Phasen der Lehrkräftebildung (Lehramtsstudium, Referendariat, berufliche Fort- und Weiterbildung) bilden die Basis für das Gelingen professionellen pädagogischen Handelns im schulischen Kontext. Beruflichen Fortbildungsangeboten in der dritten Phase kommt für den Erhalt und die Weiterentwicklung der professionellen pädagogischen Handlungskompetenz eine besondere Relevanz zu. Gerade unter dem Credo einer sich stetig wandelnden Umwelt ist die eigene Professionalisierung während der gesamten berufsbiografischen Entwicklung nie abgeschlossen und kann somit als „berufslebenslanger Prozess“ (Idel et al. 2021, S. 18) angesehen werden. Finden diese Prozesse in organisierter Form statt, lässt sich die Lehrkräftefortbildung als Bestandteil des Weiterbildungsbereichs (Deutscher Bildungsrat 1970) und damit in der erziehungswissenschaftlichen Teildisziplin der Erwachsenen- und Weiterbildung verorten.

Der Großteil der bisherigen Beiträge zur Lehrkräftefortbildung lässt sich vorwiegend in der erziehungswissenschaftlichen Teildisziplin der Schulpädagogik bzw. dem Bereich der Lehrkräftebildung verorten. Mit Blick auf die oben genannten beruflichen Anforderungen, die mit der digitalen Transformation verbunden sind, bildet die Erwachsenen- und Weiterbildung mit ihren Diskursen, Modellen und Forschungsergebnissen jedoch eine wichtige Instanz zur Bearbeitung dieser Anforderungen. Ihre Fokussierung auf das Lernen Erwachsener nach einer ersten Bildungsphase ist in unterschiedlichsten Aspekten dazu geeignet, Impulse für die Konzeption und Durchführung von Lehrkräftefortbildungen zu generieren, die einen wesentlichen Professionalisierungskontext für Lehrkräfte darstellen und einen wesentlichen Beitrag zur selektiven Modernisierung (Schrape 2021) schulischer Bildungsbereiche angesichts der Herausforderungen der digitalen Transformation leisten können. Ziel des Beitrages ist daher, die aktuellen Herausforderungen der Lehrkräftefortbildung vor dem Hintergrund der digitalen Transformation zu beschreiben und aus Perspektive der Erwachsenen- und Weiterbildung subdisziplinübergreifende Impulse für ihre Weiterentwicklung zu identifizieren. Dazu wird zunächst ein Überblick über die Lehrkräftebildung gegeben, um anschließend den aktuellen Stand der Lehrkräftefortbildung in Deutschland vor dem Hintergrund der digitalen Transformation anhand des Modells der Erwachsenen- und Weiterbildung als Mehrebenensystem nach Schrader (2019) zu betrachten. Dafür werden aktuelle Erkenntnisse zur Lehrkräftefortbildung allgemein sowie insbesondere mit Bezug zur digitalen Transformation zusammengeführt. Dabei stehen vor allem die durch die digitale Transformation veränderten Professionalisierungsbedarfe im Fokus, aber auch die damit verbundenen neuen Möglichkeiten für die Gestaltung von Fortbildungen. Die sich daraus ergebenden Herausforderungen werden aus Sicht der Erwachsenen- und Weiterbildung diskutiert.

## Die drei Phasen der Lehrkräftebildung in Deutschland

Die Lehrkräftebildung ist in Deutschland dreiphasig angelegt und durch unterschiedliche Akteurinnen und Akteure und Institutionen getragen (Cramer 2020; Terhart 2020). Allgemein wird das Lehramtsstudium als erste Phase an einer Universität oder Pädagogischen Hochschule absolviert. Je nach Lehramtsstudiengang werden die Absolventinnen und Absolventinnen dazu befähigt, in diversen Schularten bzw. Phasen zu unterrichten: Lehramt der Primarstufe, der Sekundarstufen I und II, berufliche Schulen und Förder- bzw. sonderpädagogische Lehrämter (Terhart 2020). Die Lehramtsstudiengänge sind inzwischen als Bachelor- und Masterstudiengänge organisiert. Zentrale Akteurinnen und Akteure des Hochschulstudiums in der ersten Phase sind dabei sowohl die Fachwissenschaften, die Fachdidaktiken sowie die Bildungswissenschaften, mit dem Ziel, Studierende wissenschaftlich für den Beruf als Lehrkraft zu qualifizieren.

In der zweiten Phase der Lehrkräftebildung (Referendariat) wird die Ausbildung durch die in den deutschen Bundesländern jeweils unterschiedlich benannten Studienseminare und Ausbildungsschulen sowie Mentorinnen und Mentoren im Berufseinstieg getragen. Diese Phase fokussiert am Lernort der Ausbildungsschule durch Unterrichtshospitationen und die Durchführung von eigenverantwortlichem Unterricht die Unterrichtspraxis und eine damit verbundene Handlungsorientierung. Am Lernort der Studienseminare werden aufbauend auf den Inhalten der ersten Phase pädagogische und fachbezogene Inhalte vermittelt.

Als dritte Phase wird allgemein die lebenslange berufliche Fort- und Weiterbildung von Lehrkräften subsumiert. Während unter Weiterbildungen die Angebote zum Erwerb von zusätzlichen Abschlüssen und Zertifikaten zu verstehen sind, die eine Tätigkeitserweiterung oder -änderung anstreben, sind mit Fortbildungen die Veranstaltungen gemeint, durch die Lehrkräfte Kompetenzen innerhalb des erlernten Tätigkeitsbereiches erhalten oder weiterentwickeln (Richter und Richter 2020; Rzejak und Lipowsky 2020). Da es sich bei digitalisierungsbezogenen Angeboten für Lehrkräfte vorwiegend um Fortbildungen handelt, steht dieser Bereich in diesem Beitrag im Fokus.

Die Thematik der digitalen Transformation und ihrer Folgen für die schulische Unterrichtspraxis werden schon seit längerer Zeit als ein zentrales Handlungsfeld in der Lehrkräftebildung diskutiert (Lachner et al. 2020), wobei dieser Bereich insbesondere durch die Folgen der COVID-19-Pandemie eine besondere Brisanz und Dringlichkeit entwickelt hat (Fütterer et al. 2021, Huber et al. 2020) und den Bedarf an Lehrkräftefortbildung nochmals verdeutlicht. Lehrkräftefortbildung muss auf die sich schnell verändernden Entwicklungen der digitalen Transformation reagieren und dabei Lehrkräfte auf dem aktuellen Stand halten, aber auch einem Großteil der Lehrkräfte zunächst die notwendigen Basiskompetenzen vermitteln (Eickelmann et al. 2019b). Es ist nicht davon auszugehen, dass die benötigten Kompetenzen bereits bei allen Lehrenden vorliegen, da nur etwa ein Viertel (26 %) der Lehrkräfte in Deutschland in der ICILS-2018-Studie angaben, in ihrer Ausbildung gelernt zu haben, wie man digitale Medien im Unterricht verwendet (Eickelmann et al. 2019a).

Zwar zeigt sich über verschiedene Studien hinweg, dass sich ca. 80 % der Lehrkräfte regelmäßig fortbilden (Hoffmann und Richter 2016), jedoch gibt nur ungefähr ein Drittel der Lehrkräfte in der ICILS-2018-Studie (Eickelmann et al. 2019a) für die zwei Jahre vor der Erhebung an, eine Fortbildung zur Integration digitaler Medien in Lehr- und Lernprozesse (32 %), zur fachspezifischen Verwendung digitaler Lehr- und Lernressourcen (31 %) oder einen Kurs zu Anwendungsprogrammen (26 %) besucht zu haben. Dabei liegen alle Anteile signifikant unter dem internationalen Mittelwert. Eickelmann et al. (2019b) geben zu bedenken, dass die geringen Teilnahmequoten nicht unbedingt an der fehlenden Bereitschaft der Lehrkräfte liegen müssen, sondern auch das fehlende passende Fortbildungsangebot ausschlaggebend sein könnte.

Aus den obigen Punkten resultiert ein Bedarf an entsprechenden Angeboten in der dritten Phase der Lehrkräftebildung, die möglichst passgenau an die Bedarfe der Lehrkräfte anschließen und ihre individuelle Professionalisierung unterstützen, damit sie den Herausforderungen der digitalen Transformation begegnen können. Hierbei ist zu beachten, dass es sich nicht um singuläre Bedarfskonstellationen handelt, sondern mit Blick auf die potenziell weiter zunehmende Dynamik der technologischen Entwicklungen kontinuierlich neue Professionalisierungsbedarfe generiert werden. Im folgenden Abschnitt werden daher der Stand der Lehrkräftefortbildung – auch vor dem Hintergrund der digitalen Transformation – dargestellt, Herausforderungen zur Gestaltung solch passgenauer Fortbildungen identifiziert und aus der Perspektive der Erwachsenen- und Weiterbildung diskutiert.

## Perspektiven der Erwachsenen- und Weiterbildung auf aktuelle Herausforderungen der Lehrkräftefortbildung vor dem Hintergrund der digitalen Transformation

Um Fortbildungen zu gestalten, die Lehrkräfte dabei unterstützen, Professionalisierungsprozesse zur Bewältigung der neuen Anforderungen durch die digitale Transformation zu vollziehen, sollten aus der Perspektive der Erwachsenen- und Weiterbildung diverse Aspekte berücksichtigt werden, die auf die spezifischen Anforderungen dieser Zielgruppe zugeschnitten sind. Die hier vorgestellten Facetten sind subdisziplinübergreifende Impulse, deren Umsetzung im Idealfall komplementär zu bereits existenten und wirksamen Strukturen und Abläufen zu verstehen ist.

**Figure 1:**
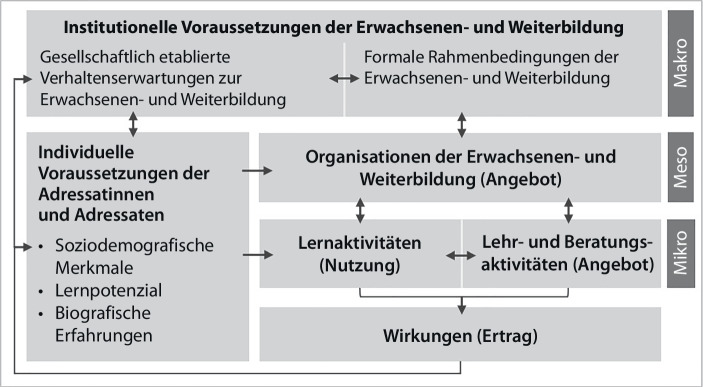

Zur Identifizierung und Strukturierung möglicher Impulse wird das Mehrebenensystem der Erwachsenen- und Weiterbildung nach Schrader (2019) herangezogen. Es stellt eine für die Erwachsenen- und Weiterbildung sensible Adaption des Angebots-Nutzungs-Modells (Helmke 2021) und eine in dieser Disziplin bewährte Heuristik dar. Durch den Einbezug dieses Modells, das alle Akteurinnen und Akteure und Strukturen des Weiterbildungsbereichs erfasst und idealtypisch auf drei Ebenen gruppiert, wird es möglich, die Konsequenzen der sektoralen Eingriffstiefe (Dolata 2011) der digitalen Transformation auf verschiedenen Ebenen der Weiterbildung zu verorten und systematisch zu reflektieren. Das Mehrebenensystem fungiert als systemtheoretisch ausgerichtetes Modell zur Erfassung von Wirkungen organisierter Erwachsenen- und Weiterbildung. Dazu werden die Input‑, Prozess- und Produktdimensionen dieses Bildungsbereichs auf diversen Strukturebenen heuristisch erfassbar (Abb. 1). Hierzu gehören die institutionellen Voraussetzungen auf der Makroebene, die Organisationen auf der Mesoebene sowie die Lernaktivitäten und Lehr- und Beratungsaktivitäten auf der Mikroebene. Diese beeinflussen kokonstruktiv die Wirkungen der Angebote. Die Wirkung im Sinne des Weiterbildungsertrages hat zudem Effekte auf die institutionellen Voraussetzungen und auf die individuellen Voraussetzungen der Adressatinnen und Adressaten, die wiederum in Beziehung zu allen anderen Ebenen des Modells stehen.

Dieser Heuristik folgend werden im Anschluss verschiedene Aspekte der Lehrkräftefortbildung in Deutschland beschrieben, die als Herausforderungen für die Umsetzung und Gestaltung bedarfsgerechter, zielgruppenorientierter und wirksamer Fortbildungen in der dritten Phase der Lehrkräftebildung gesehen werden können. Durch die Orientierung an diesen drei Attributen wird der zuvor identifizierte Bedarf an passgenauen Fortbildungen adressiert. Die diesbezüglichen Impulse gelten zum Teil für die Lehrkräftefortbildung allgemein, werden jedoch an den entsprechenden Stellen hinsichtlich ihrer spezifischen Relevanz für die Herausforderungen der digitalen Transformation ausdifferenziert.

### Makroebene – Institutionelle Voraussetzungen der Lehrkräftefortbildung

Auf der Makroebene stehen die institutionellen Voraussetzungen der Erwachsenen- und Weiterbildung im Fokus, worunter Schrader (2019) gesellschaftlich etablierte Verhaltenserwartungen zur Erwachsenen- und Weiterbildung (bspw. Weiterbildungsteilnahme als Recht oder Pflicht) sowie die formalen Rahmenbedingungen (bspw. Transparenz der Strukturen, politische Rahmenbedingungen, Qualifikationen des Personals) zusammenfasst.

In Deutschland sind Lehrkräfte rechtlich dazu verpflichtet, sich weiterzubilden, doch hat es keine beruflichen Nachteile, wenn sie dem nicht nachkommen (Richter und Richter 2020). Der Fortbildungsumfang wird nur selten von den Bundesländern quantifiziert und nur vereinzelt eingefordert, dass die Fortbildungsaktivitäten von den Lehrkräften dokumentiert werden (Rzejak und Lipowsky 2020). Auf Systemebene gibt es zudem keine Anreize für die Teilnahme an Fortbildungen (Rzejak und Lipowsky 2020), sodass die Aktivitäten primär von der jeweiligen individuellen Motivation abhängig sind. Lehrkräfte können selbstgesteuert ihre Fortbildungsaktivitäten auswählen; allerdings gibt es in der Regel keine festen Zeitkontingente, die dafür während der Dienstzeit zur Verfügung stehen (Richter und Richter 2020). In Bezug auf die oben genannten Merkmale konstatieren Richter und Richter (2020, S. 346), dass „die Voraussetzungen für eine systematische und kontinuierliche Weiterentwicklung des gesamten Lehrpersonals in Deutschland […] daher nur sehr eingeschränkt implementiert [sind].“

In Bezug auf die Fortbildungsstrukturen zeigt eine Analyse in Baden-Württemberg von Johannmeyer und Cramer (2021b), dass es eine Vielzahl an Institutionen unter dem Dach des Kultusministeriums gibt und ergänzende Angebote von Hochschulen und weiteren Trägern, wie z. B. von Kirchen organisiert werden. Die Autorinnen und Autoren konstatieren eine Unübersichtlichkeit des Fortbildungssystems mit verschiedenen Akteurinnen und Akteuren, Finanzierungsmodellen, Qualitätsvorstellungen, Themen und Wegen der Personalgewinnung. Angesichts dieser Konstellation, die weiteres Potenzial im Sinne von Transparenz beinhaltet, bietet sich die Bezugsmöglichkeit zur Erwachsenen- und Weiterbildung an. Auch hier existiert eine komplexe Struktur von Anbietern und Angeboten (Schrader 2010). Um Transparenz herzustellen, können Interessierte mittels Weiterbildungsberatung eine Orientierung erhalten (Schiersmann 2018). Analog dazu würden entsprechend zugeschnittene – auch digital gestützte – Beratungsangebote für Lehrkräfte die Möglichkeit bieten, sich zielgerichtet über digitalisierungsbezogene Fortbildungsmöglichkeiten zu informieren, das Angebot zu koordinieren und so die Rahmenbedingungen für die Nutzung von Lehrkräftefortbildungen zu verbessern. Gerade in diesem Bereich kann diese Koordination von Vorteil sein, da die Auslastung der bereits existenten Angebote vergleichsweise gering ausfällt (Johannmeyer und Cramer 2021a).

Ein weiterer wichtiger Aspekt auf Makroebene ist das Fortbildungspersonal in der Lehrkräftefortbildung, über das es kaum verlässliche Angaben gibt. Johannmeyer und Cramer (2021b) berichten, dass das Fortbildungspersonal zwar zum Teil über Ausschreibungen und Assessments ausgewählt wird, die Eignungskriterien und erforderlichen Qualifikationen jedoch weitgehend offenbleiben. Darüber hinaus gibt es keine gesicherten Angaben dazu, in welchem Ausmaß die Gestaltenden von Lehrkräftefortbildungen nach spezifischen beruflichen Standards arbeiten und sich an entsprechenden Maximen in konkreten pädagogischen Situationen orientieren. Mit Blick auf die aktuellen Diskurse in der Erwachsenen- und Weiterbildung zeigt sich jedoch die zentrale Funktion dieser Personengruppe für das Gelingen von Fortbildungen (Gieseke 2018). Die Grundlagen ihres professionellen pädagogischen Handelns bilden ein Fundus an spezifischen erziehungs- bzw. bildungswissenschaftlich fundierten Wissensbeständen sowie meist im Rahmen von akademischen Lizenzierungsverfahren erworbene Handlungskompetenzen und Methoden (Hof 2009). Zusätzlich spielen ein im Rahmen der individuellen Professionalisierung entwickeltes Rollenverständnis gegenüber sich selbst sowie gegenüber den Fortbildungsteilnehmenden und die Orientierung an wissenschaftlicher Rationalität eine zentrale Rolle (Nittel und Seltrecht 2008). Lehrende, die Fortbildungen zum allgemeinen oder fachdidaktischen Einsatz digitaler Medien im Unterricht durchführen, benötigen darüber hinaus zum einen eine hohe Expertise auf inhaltlicher bzw. fachdidaktischer Ebene und zum anderen eine hohe medienpädagogische Kompetenz zur Vermittlung dieser Inhalte. Hierzu zählen nach dem Modell medienpädagogischer Kompetenz von Lehrenden in der Erwachsenenbildung von Rohs et al. (2017) medienbezogene Feldkompetenz (z. B. Wissen über IT-Ausstattung an Schulen), medienbezogenen Einstellungen und Selbststeuerung (z. B. kritischer und reflektierter Umgang mit digitalen Medien), medienbezogener Fachkompetenz (z. B. fachdidaktischer Einsatz von digitalen Medien) und mediendidaktischer Kompetenz (z. B. didaktisch sinnvoller Einsatz digitaler Medien). Im Vergleich zum hohen Professionalisierungsrad hauptberuflicher schulischer Lehrkräfte und dessen Erforschung würde auch die weitere Verberuflichung und Professionalisierung des Fortbildungspersonals z. B. im Sinne einer (Weiter)Entwicklung geeigneter Lizenzierungsverfahren zusätzliches Potenzial für eine Qualitätssteigerung der Lehrkräftefortbildungen eröffnen.

### Mesoebene – Organisation der Lehrkräftefortbildung

Auf der Mesoebene der Erwachsenen- und Weiterbildung lassen sich alle organisationalen Tätigkeiten fassen, die zur Leitung der jeweiligen Organisation und für die Angebotsgestaltung notwendig sind. Dies umfasst bspw. die Bereiche Leitung und Management sowie die Entwicklung, Organisation und Evaluation von Angeboten und Programmen.

Um das Fortbildungsangebot zu digitalisierungsbezogenen Themen adressatengerecht organisational zu planen und zu gestalten, ist es wichtig, den Fortbildungsbedarf zu kennen. In einzelnen Studien wird dieser zwar in Bezug auf die Nutzung von digitalen Medien im Unterricht erhoben (z. B. IQB-Ländervergleich, Hoffmann und Richter 2016), jedoch gibt es in den verschiedenen Bundesländern in der Regel keine systematische Erfassung des allgemeinen und damit auch des Fortbildungsbedarfs im Bereich der digitalen Transformation (DVLfB 2018).

Betrachtet man die zeitlichen, regionalen und inhaltlichen Angebotsstrukturen der Lehrkräftefortbildung, zeigt sich, dass die Angebote überwiegend aus Halb- oder Ganztagesveranstaltungen bestehen (DVLfB 2018; Johannmeyer und Cramer 2021b). Ebenso konnten Johannmeyer und Cramer (2021b) in einer Analyse für Baden-Württemberg verdeutlichen, dass es eine signifikante regionale Ungleichverteilung des Fortbildungsangebotes gibt, wodurch Lehrkräften je nach Region ein unterschiedlich umfangreiches Angebot an Veranstaltungen zur Verfügung steht. Vor dem Hintergrund, dass eine Fortbildungsteilnahme von Lehrkräften, insbesondere an umfassenderen Angeboten, vermutlich häufig zeitlich schwierig und das Angebot regional unterschiedlich verteilt ist, könnten zeit- und ortsunabhängige Onlineformate die Fortbildungsteilnahme für manche Lehrkräfte erleichtern. Diese Formate könnten angesichts der langfristigen Effekte der digitalen Transformation bspw. im Bereich des technischen Wandels und des daraus resultierenden Professionalisierungsbedarfs für Lehrkräfte eine komplementäre oder zumindest eine kompensatorische Funktion im Verhältnis zu Präsenzveranstaltungen ausfüllen. Es ist prospektiv davon auszugehen, dass nach den pandemiebedingten Entwicklungen der letzten Jahre synchrone und asynchrone Onlineangebote zumindest zum Teil bestehen bleiben und dadurch ein auf die oben genannten Aspekte bezogen niedrigschwelligeres Fortbildungsangebot entstehen kann.

In Bezug auf die inhaltliche Struktur stehen die Fortbildungsangebote häufig für sich alleine, ohne in ein inhaltliches Gesamtkonzept eingebettet zu sein und weisen damit eine geringe Kohärenz auf, wodurch ein zielgerichteter Kompetenzaufbau nur schwer möglich ist (DVLfB 2018; Rzejak und Lipowsky 2020). Dieser Umstand ist auch durch die COVID-19-Pandemie deutlich zutage getreten. Im Zuge der Pandemie gab es verschiedene Fortbildungsangebote, die jedoch im „Notfallmodus“ erstellt wurden, obwohl strukturierte, systematische Angebote notwendig gewesen wären. Und auch schon vor der Pandemie zeigt eine Analyse der Lehrkräftefortbildungen in Baden-Württemberg von Schmidt-Hertha (2020), dass die inhaltliche Ausrichtung von Fortbildungen mit Bezug zu digitalen Medien häufig auf medienpraktisches Wissen fokussiert zu sein scheint, wie z. B. die Anwendung einzelner digitaler Tools oder Plattformen, und weniger grundlegende medienpädagogische Kompetenzen systematisch vermittelt werden. Zu ähnlichen Ergebnissen kommt eine Studie von Diepolder et al. (2021), in der das Fortbildungsangebot zu digitalen Medien mit Fokus auf fachdidaktisch-naturwissenschaftliche Fortbildungsangebote in zwölf Bundesländern untersucht wird.

Eine Möglichkeit, um die Attraktivität einzelner Lehrkräftefortbildungen bereits in der Planungsphase zu verbessern und so die Teilnahmequote zu erhöhen, stellt die gezielte Ansprache der Zielgruppe dar, wobei die Teilnahmemotivation der Lehrkräfte über entsprechende Marketinginstrumente, wie die Gestaltung des Angebots, der Kommunikation, der Distribution und der „Gegenleistungsgestaltung“, wie materielle Kosten (z. B. Preis) und immaterielle Kosten (z. B. Zeit) (Schöll 2018) adressiert werden kann. Hierfür ist jedoch zunächst eine strukturierte, zielgruppenfokussierte Bedarfsanalyse notwendig, um die Ansprache passgenau zu gestalten. Schulze-Vorberg et al. (2021) empfehlen in ihrer Studie u. a. Fortbildungen mit verschiedenen Anforderungsniveaus anzubieten (Grundlagen, Angebote für Fortgeschrittene), in der Ausschreibung sowohl einen hohen inhaltlichen Bezug zur Unterrichtspraxis mit konkreten fachdidaktischen Angeboten als auch die Möglichkeit zum kollegialen Austausch in den Fortbildungen deutlich zu machen sowie bei den Angeboten die ort- und zeitunabhängigen Potenziale von digitalen Formaten verstärkt zu nutzen. Durch eine solche – auf aktuellen Bedarfsanalysen basierende – zielgruppenorientierte Bewerbung der Angebote können die Chancen verbessert werden, um möglichst viele Lehrkräfte für Fortbildungen zu gewinnen und die Nachfrage sowie Auslastung der digitalisierungsbezogenen Angebote zu erhöhen.

### Mikroebene – Lehr-Lernprozesse im Kontext von Lehrkräftefortbildung

Auf der Mikroebene verortet Schrader (2019) die Lehr-Lernprozesse, wobei zum einen die Nutzung dieser Angebote im Sinne von konkreten Lernaktivitäten der Lernenden fokussiert ist, was etwa eine Erfassung von Weiterbildungsteilnahme, Lernmotivation und kognitiver Beteiligung ermöglicht. Zum anderen werden die Lehr- und Beratungsaktivitäten betrachtet, wobei exemplarisch Aspekte wie die Vorbereitung, Durchführung und Evaluation von Lehr-Lernprozessen oder die Berücksichtigung erwachsenenpädagogischer Prinzipien hervortreten.

Aus verschiedenen Studien zur Wirksamkeit von Lehrkräftefortbildungen lassen sich verschiedene Wirksamkeitsprädiktoren in Bezug auf die Gestaltung der Lehr-Lernprozesse festhalten (DVLfB 2018, S. 132 ff.). Neben der inhaltlichen Orientierung an Befunden der Unterrichts- und Lehr-Lernforschung, dem Blick auf fachbezogene Lernprozesse von Schülerinnen und Schülern, den Möglichkeiten für Coaching und Feedback und der Gelegenheit, die Wirksamkeit des eigenen Handelns zu erleben, können auch die Ermöglichung und Stärkung einer intensiven kollegialen Zusammenarbeit sowie eine sinnvolle Gestaltung der Fortbildungszeit durch die Verbindung von Input, Erprobung und Reflexion zu diesen Prädiktoren gezählt werden. Die Autorinnen und Autoren geben jedoch zu bedenken, dass die zusammengetragenen Studien Fortbildungen untersuchen, die über einen längeren Zeitraum stattfinden und damit umfangreichere Lernaktivitäten ermöglichen. Über die Prädiktoren für die Wirksamkeit der oben genannten häufig vorkommenden halb- oder ganztägigen Veranstaltungen lassen sich somit nur begrenzt Aussagen treffen.

Für eine transferförderliche Gestaltung einer Fortbildung ist unter anderem das Transferdesign ausschlaggebend (z. B. Orientierung an allgemeinen didaktischen Prinzipien, realitätsnahe Übungen, Follow-up-Termine) (Kauffeld 2016). Aus der Forschung zu Lehrkräftefortbildungen wissen wir zudem, dass wirksame Fortbildungen idealerweise die Elemente Vermittlung, Erarbeitung, Erprobung, Rückmeldung und Reflexion enthalten (Lipowsky 2014). Diese Elemente können jedoch nur bedingt in den überwiegend kürzeren Fortbildungsformaten umgesetzt werden, da es zeitbedingt wenig Möglichkeiten für Anwendung und Transfer gibt. Das persönliche Interesse an der Auseinandersetzung mit neuen Themen kann in diesem Kontext als starke Motivation von Lehrkräften zur Nutzung von Fortbildungen identifiziert werden (Cramer et al. 2019). Dieser Umstand impliziert zum einen die Anforderung, sich bereits in der Konzeptionsphase des Angebots im Sinne der Zielgruppenorientierung (v. Hippel et al. 2019) mit dem Wissensstand und den antizipierten Lernbedürfnissen der potenziellen Teilnehmenden zu beschäftigen. Zum anderen eröffnet sich daraus die Anforderung, auch während der Durchführung des Angebots auf die Interessen der Teilnehmenden einzugehen und so das erwachsenenpädagogische Prinzip der Teilnehmendenorientierung umzusetzen.

Um die Lehr-Lernprozesse zu unterstützen, ist auch der Einbezug des Arbeitsumfeldes wichtig (Blume et al. 2010; Salas et al. 2012). Bedeutsam sind zum einen die Unterstützung durch Kolleginnen und Kollegen und Vorgesetzte, zum anderen aber z. B. auch die Möglichkeit, das Gelernte tatsächlich anzuwenden. Letzteres kann schon an einer nicht ausreichenden technischen Infrastruktur (WLAN, IT-Ausstattung, Verfügbarkeit von Lernmanagementsystemen) scheitern, die an deutschen Schulen unter dem internationalen Durchschnitt liegt (Eickelmann et al. 2019a).

Neben der didaktischen Gestaltung sind auch die Inhalte der Fortbildungen von Bedeutung. In den letzten zwei Jahrzehnten sind verschiedene Kompetenz- und Wissensmodelle entwickelt worden, die angesichts der Komplexität der digitalen Transformation Impulse für die Professionalisierung von Lehrkräften setzen können. Kompetenzrahmen wie der Europäische Rahmen für die Digitale Kompetenz von Lehrenden (DigCompEdu, Redecker 2017) oder das DPACK-Modell (Döbeli Honegger 2021) könnten dabei eine Basis für die inhaltliche Systematisierung und Gestaltung von Fortbildungsangeboten sein. Im DPACK-Model werden das TPACK-Modell (Mishra und Koehler 2006) und das Dagstuhl-Dreieck (Gesellschaft für Informatik e. V. 2016) miteinander verbunden. Es verweist darauf, dass es sowohl pädagogische als auch inhaltliche sowie digitale Kompetenzen benötigt, um Unterricht in einer Kultur der Digitalität zeitgemäß zu gestalten (Döbeli Honegger 2021). Im Vergleich zum TPACK-Modell wird von Kompetenzen und nicht mehr nur von Wissen gesprochen. Das Technikwissen wird durch Digitalitätskompetenz ersetzt und ergänzt dadurch die technologische Perspektive, die Anwendungsperspektive und die gesellschaftlich-kulturelle Perspektive des Dagstuhl-Dreiecks. Die bisherige Fortbildungslandschaft zeichnet sich im Kontrast zu den beschriebenen Ansätzen oft durch Angebote aus, die vorwiegend die technische Seite der digitalen Transformation, bspw. die Handhabung von Technologien, thematisieren (Schmidt-Hertha 2020). Dahingehend sollten Fortbildungen für Lehrpersonen die Vermittlung von handlungsorientiertem Wissen bzw. von Kompetenzen unter Einbezug aller oben genannter Facetten fokussieren.

### Individuelle Voraussetzungen für die Teilnahme an Lehrkräftefortbildung

Zu den individuellen Voraussetzungen der Adressatinnen und Adressaten gehören deren soziodemografische Merkmale, ihre biografischen Erfahrungen sowie ihr jeweiliges Lernpotenzial. Das Wissen über diese Merkmale stellt eine zentrale Voraussetzung für die zielgruppen- und teilnehmendenorientierte Gestaltung von Fortbildungsangeboten auf der Meso- und Mikroebene dar.

Vor dem Hintergrund der digitalen Transformation zählen zu diesen Merkmalen z. B. die Vorerfahrung mit digitalen Medien oder die Einstellung gegenüber der Verwendung von digitalen Medien im Unterricht. Bisher verwenden Lehrkräfte in Deutschland digitale Medien im internationalen Vergleich eher in einem geringen Umfang, sehen aber durchaus das lernförderliche Potenzial digitaler Medien (Eickelmann et al. 2019a). Ergebnisse aus der Erwachsenen- und Weiterbildungsforschung legen in diesem Kontext die Orientierungskraft eines medialen Habitus nahe (Bolten-Bühler 2021). Dieser bestimmt die Affinität bzw. Grundeinstellung der jeweiligen Person gegenüber digitalen Medien und kann je nach Ausmaß der Vorerfahrungen und ihrer biografischen Kontextualisierung zu einer eher funktionalen Grundhaltung oder einer zumindest ambivalenten Positionierung ihnen gegenüber führen, was wiederum die eigene medienpädagogische Professionalisierung beeinflussen kann.

Verbunden mit der in Kapitel 2 thematisierten Fortbildungsteilnahme lassen sich auf Ebene der Adressatinnen und Adressaten auch mögliche Gründe für die Nichtteilnahme an Fortbildungsangeboten identifizieren. Erkenntnisse aus der Erforschung von Sekundarlehrkräften (Richter et al. 2018) und Lehrkräften in Baden-Württemberg (Müller 2021) bestätigen den Diskussionsstand der Erwachsenen- und Weiterbildung zur Vielfalt von Weiterbildungsbarrieren (Kuwan und Seidel 2013): Das genuine Desinteresse an formalen Lernkontexten bzw. die dahinterstehenden Lernpräferenzen und allgemeinen Weiterbildungseinstellungen, die Herausforderungen der zeitlichen Organisation, das Ausmaß der finanziellen Aufwendungen, ein schwieriges berufliches Umfeld sowie hemmende persönliche Lebenssituationen bilden zentrale Barrieren für Erwachsene in unterschiedlichen Lebenslagen und beruflichen Konstellationen. Schulze-Vorberg et al. (2021) befragten Lehrkräfte zu Hinderungsgründen für die Teilnahme an Fortbildungen zu digitalen Medien in den letzten zwölf Monaten.

Zur Begründung der Nichtteilnahme an medialen Fortbildungen gaben die Lehrkräfte insbesondere an, dass sie andere Themenbereiche als wichtiger eingestuft hätten, dass die digitalen Fortbildungsangebote ungeeignet gewesen oder zu unpassenden Zeiten stattgefunden hätten und dass die persönliche Energie zur Teilnahme gefehlt hätte. Darüber hinaus wurde der fehlende Bezug der Angebote zur Schulrealität und zur Fachdidaktik als Hinderungsgrund angegeben sowie allgemein der fehlende praktische Nutzen von Fortbildungen. Einige Lehrkräfte gaben als Grund für eine Nichtteilnahme an, dass sie sich ohne Fortbildung auf dem Laufenden halten könnten, wobei dies vor allem für Lehrkräfte zutraf, die ihr technologisches und technologisch-pädagogisches Wissens sowie ihre digitale Selbstwirksamkeitserwartung als besonders hoch einschätzen. Für gelingende Lehrkräftefortbildungen lässt sich daraus der Impuls ableiten, die Teilnahmegründe der Zielgruppe nach Möglichkeit systematisch zu eruieren und das Angebot so zu gestalten, dass Teilnahmebarrieren möglichst vermieden werden.

### Wirkungen von Lehrkräftefortbildung

Die Wirkungen der Erwachsenen- und Weiterbildung auf Individuen, Organisationen und Gesellschaft werden als kokonstruktives Handlungsresultat von Akteurinnen und Akteuren unterschiedlicher Systemebenen erfassbar. Die Wirkungen lassen sich auf vier Evaluationsebenen erfassen (Kirkpatrick 1998): Reaktionen (z. B. Zufriedenheit mit der Fortbildung), Lernerfolge (z. B. Wissenszuwachs über mediendidaktische Prinzipien), Transfererfolge (z. B. der didaktisch sinnvolle Einsatz digitaler Medien im Unterricht) und Ergebnisse (z. B. ein verbesserter Lernerfolg von Schülerinnen und Schüler durch eine verbesserte Unterrichtsqualität).

Auf Ebene der Bundesländer gibt es häufig keine differenzierten Statistiken oder Evaluationen des Fortbildungsangebotes (DVLfB 2018), die jedoch notwendig wären, um das Angebot evidenzbasiert weiterzuentwickeln. In Bezug auf den Ertrag von Lehrkräftefortbildungen ist das zentrale Ziel in der Regel der Transfer des Gelernten in die Unterrichtspraxis. Auch wenn es bereits einige Erkenntnisse zur Wirksamkeit von Lehrkräftefortbildungen gibt (Lipowsky 2014; DVLfB 2018), zeigt sich weiterer Forschungsbedarf, um Lehrkräftefortbildungen evidenzbasiert zu gestalten. Besonders anschlussfähig ist hier die Weiterbildungs- und Transferforschung, die in den letzten Jahrzehnten empirisch fundierte Erkenntnisse darüber hervorgebracht hat, welche Faktoren transferförderlich sind (z. B. Arthur et al. 2003; Burke und Hutchins 2007; Blume et al. 2010; Salas et al. 2012; Ford et al. 2018). Aufbauend auf dem Transfermodell von Baldwin und Ford (1988) gibt es drei Bereiche, die ausschlaggebend für den Fortbildungserfolg sind und in den vorherigen Abschnitten bereits thematisiert wurden: die Teilnehmendenmerkmale, das Arbeitsumfeld und die Gestaltung der Fortbildung. Die Gestaltung hängt zudem mit einem weiteren, vierten Faktor zusammen, der professionellen Handlungskompetenz des durchführenden Lehrpersonals (Burke und Hutchins 2008; Bonnes et al. 2019). Es fehlen jedoch, wie bereits beschrieben, die notwendigen Erkenntnisse zum Lehrpersonal in der Lehrkräftefortbildung, wobei eine Professionalisierung des Weiterbildungspersonals von großer Bedeutung auch für die Qualität des digitalisierungsbezogenen Fortbildungsangebotes wäre. Einen ersten Schritt in diese Richtung bildet das GRETA-Kompetenzmodell 2.0, in dem die professionellen Handlungskompetenzen Lehrender mit Bezug zu digitalen Kompetenzen erweitert wurden (Alberti et al. 2022).

Die Wirkungsforschung in der Lehrkräftefortbildung sollte sich daher an den bestehenden Modellen und Erkenntnissen der Weiterbildungs- und Transferforschung orientieren, um Prädiktoren auf Ebene der Teilnehmenden und des Fortbildungsdesigns sowie auf Ebene des Arbeitsumfeldes und des Weiterbildungspersonals zu untersuchen. Auf dieser Basis können Erkenntnisse für die zukünftige Gestaltung von wirksamen Fortbildungen generiert werden.

## Zusammenfassung und Ausblick

Im Beitrag wurden die aktuellen Herausforderungen in der Lehrkräftefortbildung vor dem Hintergrund der sich durch die digitale Transformation verändernden Anforderungen dargestellt und entlang des Mehrebenensystems nach Schrader (2019) aus der Perspektive der Erwachsenen- und Weiterbildung diskutiert. Ziel war es, Impulse für die Weiterentwicklung der Lehrkräftefortbildung zu geben, um die Konzeption und Umsetzung von zielgruppenorientierten und wirksamen Fortbildungsangeboten und letztlich verbesserte Rahmenbedingungen für die Professionalisierungsprozesse der Lehrkräfte zu fördern.

Zu diesem Zweck wurden zunächst die drei Phasen der Lehrkräftebildung beschrieben, um die Verbindung zwischen ihrer dritten Phase und der Erwachsenen- und Weiterbildung zu verdeutlichen. Daran anknüpfend wurden aktuelle Herausforderungen bei der Gestaltung bedarfsgerechter, zielgruppenorientierter und wirksamer Fortbildungsangebote identifiziert und anschließend aus Perspektive der Erwachsenen- und Weiterbildung diskutiert. Es wurde deutlich, dass Herausforderungen nicht nur in einem Bereich bestehen, sondern die sektorale Eingriffstiefe auf allen Systemebenen zu registrieren ist. Zusammenfassend lassen sich verschiedene Aspekte auf den einzelnen Ebenen benennen, die für die Lehrkräftefortbildung vor dem Hintergrund der digitalen Transformation von Bedeutung sind.

Auf der Makroebene finden sich neben suboptimalen Anreizstrukturen und unzureichenden Monitoringinstrumenten für den Fortbildungsbedarf auch fehlende Freiräume während der Dienstzeit sowie eine geringe Überprüfung der regelmäßigen Fortbildungsteilnahme. Deutlich wird auch eine ausbaufähige Transparenz des Fortbildungssystems und seiner Strukturen. Ergänzt wird diese Konstellation durch fehlende Daten bzw. Konzepte zur Professionalisierung des Fortbildungspersonals, wobei dies jedoch einen wichtigen Gelingensfaktor für wirksame Fortbildungen allgemein als auch für digitalisierungsbezogene Fortbildungen darstellt, da neben erwachsenenpädagogischer Kompetenz auch die Anforderungen an die medienpädagogische Kompetenz besonders hoch sind.

Auf der Mesoebene zeigt sich ein deutliches Desiderat für eine strukturierte und systematische Bedarfsanalyse, um Fortbildungsangebote zu digitalisierungsbezogenen Themen adressatengerecht zu planen und zu gestalten. Weitere Optimierungsmöglichkeiten werden mit Blick auf die inhaltliche, zeitliche und regionale Struktur der Angebote deutlich, um Lehrkräften ein Fortbildungsangebot zu machen, das medienpädagogische Kompetenz systematisch vermittelt, sich mit den Arbeitszeiten vereinbaren lässt und regional unabhängig leicht zugänglich ist, z. B. über digital gestützte Angebote und Fortbildungen.

Für die Mikroebene kann der Impuls generiert werden, dass sich sowohl die Gestaltung von Lehrkräftefortbildungen an den Ergebnissen der Wirksamkeitsforschung in der Lehrkräftefortbildung als auch an der Transfer- und Weiterbildungsforschung orientieren sollte. Um Lehrkräfte auf die Herausforderungen der digitalen Transformation vorzubereiten, benötigt es auf der inhaltlichen Ebene nicht nur Fortbildungen, die die Mediennutzung adressieren, sondern systematisch eine grundlegende medienpädagogische Kompetenz vermitteln, angelehnt an umfassende Kompetenzmodelle wie z. B. DigCompEdu (Redecker 2017) oder dem DPACK-Modell (Döbeli Honegger 2021).

Aufseiten der Adressatinnen und Adressaten wird vor allem mit Blick auf die digitale Transformation und den Einsatz digitaler Medien im Unterricht die Bedeutung der individuellen Voraussetzungen deutlich, z. B. in Bezug auf Vorerfahrungen und Einstellungen oder den Einfluss eines medialen Habitus. Erkenntnisse über diese individuellen und je nach Fortbildungskontext variierenden Voraussetzungen der Adressatinnen und Adressaten sind von zentraler Bedeutung, da sie Konsequenzen für die Konfiguration der Makro‑, Meso- und der Mikroebene haben.

Auf Ebene der Wirkungen von Fortbildungen gilt es zunächst, eine belastbare Datenbasis zu generieren, um Angebote evidenzbasiert zu gestalten. Hierfür ist es notwendig, dass zukünftige Angebote systematisch evaluiert werden und zwar nicht nur auf Ebene der Zufriedenheit, sondern auch auf Ebene des Lernerfolgs, des Transfererfolgs und letztlich auch, ob die Wirkung der Fortbildung auch die Ergebnisebene erreicht (Kirkpatrick 1998), z. B. durch eine Steigerung der Unterrichtsqualität und einen dadurch erhöhten Lernerfolg von Schülerinnen und Schüler.

Zusammenfassend zeigt sich, dass aus einer systemtheoretischen Perspektive auf den verschiedenen Ebenen und Bereichen der Lehrkräftefortbildung erkennbares Optimierungspotenzial besteht. Angesichts dieser Konstellation und vor dem Hintergrund der Expertise der Lehrkräftebildung in der dritten Phase und der Expertise der Erwachsenen- und Weiterbildung wurden im vorliegenden Beitrag Schnittpunkte für subdisziplinübergreifende Forschungsfelder und Impulse für die Weiterentwicklung der Fortbildungspraxis aufgezeigt.

Gleichwohl bleibt festzuhalten, dass durch solche übergreifenden Impulse für die Lehrkräftefortbildung keineswegs alle der beschriebenen Herausforderungen bearbeitet werden können. Darüber hinaus wurde in diesem Beitrag das informelle arbeitsplatzbezogene Lernen ausgeklammert, das gerade am Anfang der Pandemie eine wichtige Rolle für den Ausbau der Kompetenzen im Umgang mit digitalen Medien zur zeitnahen Bearbeitung der Anforderungen der digitalen Transformation gespielt hat (Dreer und Kracke 2021). Die oben genannten Ergebnisse zu den Hinderungsgründen für eine Fortbildungsteilnahme von Schulze-Vorberg et al. (2021) fungieren als Hinweis darauf, dass diese Lernform insbesondere von Lehrkräften mit bereits hohen digitalisierungsbezogenen Kompetenzen genutzt wird. Mit Blick auf die Anforderungen professionellen pädagogischen Handelns sind diese informellen Lernprozesse keineswegs zu vernachlässigen. Jedoch sollten sie nicht den Standard der beruflichen Fortbildung bilden, da die notwendigen systematischen und zielgerichteten Professionalisierungsprozesse entsprechend gestaltete Fortbildungsangebote benötigen. Die Erwachsenen- und Weiterbildung kann hier Impulsgeberin sein, um die Lehrkräftefortbildung aus einer subdisziplinübergreifenden Perspektive heraus auf System‑, Organisations‑, Angebots- und Professionsebene weiterzuentwickeln.
